# Endosonography-Guided Biliary Drainage with One-Step Placement of a Newly Developed Fully Covered Metal Stent Followed by Duodenal Stenting for Pancreatic Head Cancer

**DOI:** 10.1155/2010/426534

**Published:** 2010-10-19

**Authors:** Kei Ito, Naotaka Fujita, Yutaka Noda, Go Kobayashi, Takashi Obana, Jun Horaguchi, Shinsuke Koshita, Yoshihide Kanno, Takahisa Ogawa, Yuhei Kato, Yasunobu Yamashita

**Affiliations:** Department of Gastroenterology, Sendai Medical Center, 5-22-1, Tsurugaya, Miyagino-ku, Sendai, Miyagi 983-0824, Japan

## Abstract

An 83-year-old man was admitted to our department, presenting with jaundice, fever, and nausea. CT revealed a pancreatic head tumor with duodenal invasion. Endoscopic transpapillary biliary drainage was unsuccessful due to stenosis at the second portion of the duodenum and tumor invasion to the papilla of Vater. Using a convex linear array echoendoscope, a fully-covered metal stent was placed across the puncture tract to bridge the duodenum and the bile duct. After improvement of jaundice, a duodenal metal stent was placed across the stricture of the duodenum. No procedure-related complications occurred. Neither migration nor obstruction of the two stents was observed during the three months followup period. Combination of ESBD using a fully covered metal stent and duodenal stenting is a feasible technique and possibly a less invasive treatment option for malignant biliary and duodenal obstruction compared to surgery.

## 1. Introduction

 Transpapillary endoscopic biliary drainage (EBD) is the standard treatment in patients with biliary obstruction. However, it is not always possible to perform transpapillary biliary decompression, especially in patients with duodenal stenosis or difficult cannulation of the bile duct. We herein report a case who underwent endosonography-guided biliary drainage with one-step placement of a newly developed fully-covered metal stent followed by duodenal stenting for pancreatic head cancer.

## 2. Case Report

**Figure 1:**
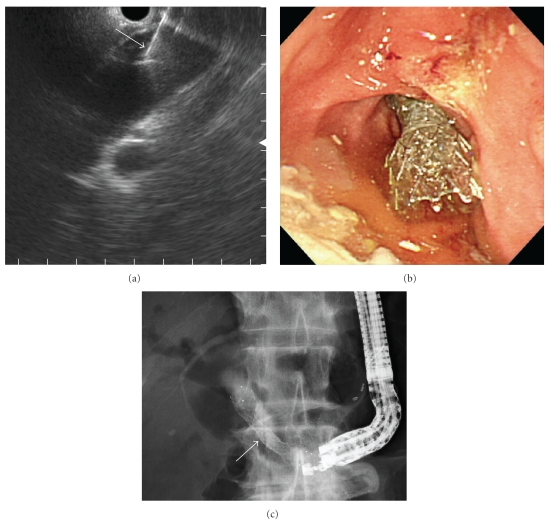
Endosonography-guided biliary drainage ((a) endosonography; (b) duodenoscopy; (c) fluoroscopy). Using a convex linear-array echoendoscope, puncture of the extrahepatic bile duct via the duodenal bulb was performed with a 19G needle (arrow) (a). A fully-covered metal stent (c, arrows) (covered ZEOSTENT), 6 cm in length and 1 cm in diameter, was placed via the puncture tract (b, c).

**Figure 2:**
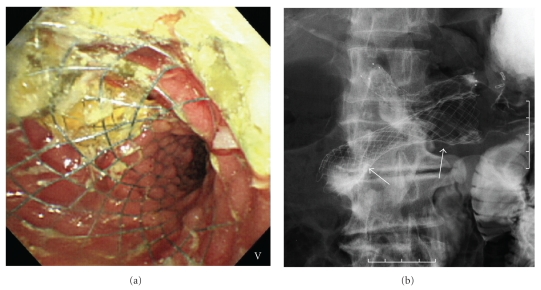
Duodenal stenting ((a) endoscopy; (b) fluoroscopy). Using a duodenal scope, a duodenal metal stent (b, arrows) (WallFlex duodenal stent), 6 cm in length and 22 mm in diameter, was placed across the stricture (a, b).

 An 83-year-old man, who had previously undergone partial gastrectomy with Billroth-I reconstruction due to gastric cancer, was admitted to our department, presenting with jaundice, fever, and nausea. Laboratory data showed an elevation of hepatobiliary enzyme and C-reactive protein. CT revealed a pancreatic head tumor with duodenal invasion. He was able to take only liquid food orally due to duodenal stenosis. It was necessary to perform biliary drainage first due to acute cholangitis. Endoscopic transpapillary biliary drainage was unsuccessful due to stenosis at the second portion of the duodenum and tumor invasion to the papilla of Vater. Using a convex linear array echoendoscope (GF-UCT240: Olympus Medical Systems, Co., Ltd., Tokyo, Japan), puncture of the extrahepatic bile duct via the duodenal bulb was performed with a 19G needle after obtaining the informed consent from the patient (Figure 1(a)). Following dilation of the puncture tract with a balloon catheter, 4 mm in diameter, a fully-covered metal stent (covered ZEOSTENT: Zeon Medical Inc., Tokyo, Japan), 6 cm in length and 1 cm in diameter, was placed across the puncture tract to bridge the duodenum and the bile duct (Figures 1(b) and 1(c)). After improvement of jaundice, using a duodenal scope (TJF 260V: Olympus Medical Systems), a duodenal metal stent (WallFlex duodenal stent: Microvasive, Boston Scientific Corp., Natick, MA), 6 cm in length and 22 mm in diameter, was placed across the stricture of the duodenum (Figure 2). No procedure-related complications occurred. He was able to take food orally and was discharged. Neither migration nor obstruction of the two stents was observed during the three months follow-up period.

## 3. Discussion

 Metal stents with a large diameter can offer longer stent patency than plastic stents for malignant biliary stricture [1]. Transpapillary endoscopic biliary drainage is the standard treatment in such patients. However, it is not always possible to perform transpapillary biliary decompression, especially in patients with duodenal stenosis or difficult cannulation of the bile duct. Endosonography-guided biliary drainage (ESBD) has been developed as a new biliary drainage technique to overcome such instances [2–17].

 ESBD was first performed by Giovannini et al. [2] in 2001 for a patient with pancreatic cancer at unsuccessful ERCP. They punctured the extrahepatic bile duct via the duodenum under ES guidance and succeeded in placement of a 10 F plastic stent across the bile duct and the duodenum. Kahaleh et al. [3] reported the largest case series (*n* = 23) of patients who had undergone ESBD, with a technical success and complication rate of 91% and 17%, respectively. Horaguchi et al. [4] reported 16 cases of difficult transpapillary endoscopic drainage, with a technical success and complication rate of 100% and 6%, respectively.

 Although a metal stent covered by a membrane contributes to the prevention of bile peritonitis as well as stent occlusion due to tissue hyperplasia following ESBD, fully covered metal stents entail a risk of stent migration. Recently, Park et al. [10] reported a prospective study of a fully covered metal stent in 14 cases of malignant biliary obstruction with unsuccessful ERCP. In their study, stent migration occurred in one patient. 

 We performed one-step placement of a newly developed fully-covered metal stent which has several unique characteristics. One is the shape of the stent after full expansion. It has a wavy contour with an uneven outer surface, which expectedly contributes to prevention of stent migration. A very low shortening rate is another characteristic, which facilitates accurate deployment of the stent. Stent migration was not observed during the follow-up period in the present case.

 Cases of biliopancreatic malignancy often develop not only obstructive jaundice but also duodenal stenosis in its advanced stage. Duodenal stenting has been reported to be useful for malignant duodenal stenosis [18]. Performance of duodenal stenting first may expand the chance for successful endoscopic transpapillary biliary drainage when the papilla of Vater has not been involved in cancer invasion. However, if apparent acute cholangitis exists, biliary drainage should be considered first, as in the present case.

 In conclusion, combination of ESBD using a fully covered metal stent and duodenal stenting is a feasible technique and possibly a less invasive treatment option for malignant biliary and duodenal obstruction compared to surgery. Further accumulation of such cases and adequate comparative studies are awaited for the assessment of the effectiveness of this technique.
